# Fetal NT-proBNP levels and their course in severe anemia during intrauterine treatment

**DOI:** 10.1007/s00404-023-07006-8

**Published:** 2023-03-26

**Authors:** Pauline Siebers, Ulrich Gembruch, Waltraut Maria Merz, Florian Recker, Andreas Müller, Brigitte Strizek, Annegret Geipel, Christoph Berg, Eva Christin Weber

**Affiliations:** 1 Department of Obstetrics and Prenatal Medicine, University Hospital Bonn, Bonn, Germany; 2 Department of Neonatology and Pediatric Intensive Care, University Hospital Bonn, Bonn, Germany; 3 Division of Prenatal Medicine, Gynecological Ultrasound and Fetal Surgery, Department of Obstetrics and Gynecology, University Hospital Cologne, Cologne, Germany

**Keywords:** Fetal anemia, Hydrops fetalis, NT-proBNP, Fetal heart, Cardiac failure, Intrauterine transfusion

## Abstract

**Purpose:**

In adults and fetuses, N-terminal pro-B-type natriuretic peptide (NT-proBNP) is a marker of cardiac failure and myocardial remodelling. We examined the effect of anemia and intrauterine transfusion (IUT) on NT-proBNP concentrations in fetuses with anemia and established gestational age-dependent reference values of a control group.

**Methods:**

We analyzed NT-proBNP levels in anemic fetuses that underwent serial intrauterine transfusions (IUT), focusing on different causes and severity of anemia and comparing the results to a non-anemic control group.

**Results:**

In the control group, the average NT-proBNP concentration was 1339 ± 639 pg/ml, decreasing significantly with increasing gestational age (*R* = − 74.04, *T* = − 3.65, *p* = 0.001). Subjects had significantly higher NT-proBNP concentrations before initiation of IUT therapy (*p* < 0.001), showing fetuses with parvovirus B19 (PVB19) infection having the highest concentrations. Hydropic fetuses also showed an increased NT-proBNP concentration compared to non-hydropic fetuses (*p* < 0.001). During the course of therapy, NT-proBNP concentration before subsequent IUT decreased significantly from pathologically high levels, while MoM-Hb and MoM-MCA-PSV remained pathological.

**Conclusion:**

NT-pro BNP levels in non-anemic fetuses are higher than in postnatal life, decreasing with ongoing pregnancy. Anemia is a hyperdynamic state and its severity correlates with circulating NT-proBNP levels. Highest concentrations occur in fetuses with hydrops and with PVB19 infection, respectively. Treatment by IUT leads to a normalisation of NT-proBNP concentrations, so the measurement of its levels may be useful in therapy monitoring.

## What does this study add to the clinical work

**Table:** 

NT-proBNP can be a useful marker to monitor cardiac stress in anemic fetuses before and during intrauterine transfusions.	

## Introduction

Severe anemia of the fetus is defined as a low cord blood concentration of hemoglobin (Hb) more than 7–10 g/dl below the mean for gestational age [1]. Causes can be immunological (alloimmunization), such as Rhesus incompatibility, or non-immunological, such as PVB19 infections. Ultrasound findings signaling hydrops fetalis (skin edema, ascites, hydrothorax, cardiomegaly, placentomegaly, polyhydramnios) and an increased peak systolic velocity of the middle cerebral artery (MCA-PSV), measured by Doppler ultrasound, lead to the diagnosis [2]. Fetal anemia causes increased cardiac output and hyperdynamic circulatory adaptions, such as increased myocardial stretching, wall stress and filling pressures that cause cardiac remodeling and cardiomegaly. In fetuses with severe anemia, a marked increase in coronary perfusion can be demonstrated by Doppler echocardiography [3]. Coronary perfusion of the hypertrophic myocardium may increase up to fivefold, but when those adapting mechanisms are exhausted, myocardial ischemia occurs. Severe fetal anemia can cause heart failure with hydrops, as the fetal compartiments react sensitively to circulatory stress and intrauterine demise becomes imminent [4].

N-terminal pro-B-type (NT-proBNP) is a marker of cardiac dysfunction and myocardial remodeling, known in adult [5], as well as fetal medicine [6]. Its circulating levels correlate with myocardial wall stress, cardiac workload and an increased central venous pressure [7]. In the fetus, the natriuretic system, which regulates blood pressure by diuresis and vasodilatation, starts at mid-gestation [8]. Various studies presented normal values for newborns and children [9] but only very few report on the circulating concentration prenatally [10]. Increased NT-proBNP levels have been presented in fetuses with cardiovascular dysfunctions, such as structural cardiac malformations [11], as well as in fetuses with anemia [6], but also in fetuses with urinary tract malformations [12] or severe growth restriction [13]. Few studies have presented NT-proBNP values during treatment with intrauterine transfusions [6, 14].

Having doubled our numbers over the past decade, in this study, we aimed to update information on fetal NT-proBNP values, stating normal values during pregnancies of fetuses without increased cardiac load, and comparing those to anemic fetuses during treatment with serial intrauterine transfusions.

## Methods

This retrospective study included all fetuses receiving intrauterine transfusion (IUT) (subjects) between March 2009 and October 2020 and fetuses that underwent fetocide by intravascular injection of potassium chloride, without a suspected disease that could influence fetal NT-proBNP levels (controls) between January 2017 and October 2020 at the tertiary center for Prenatal Medicine of the University of Bonn. In our center, we perform about 50 IUTs and 100 fetocides annually. Ethical approval was achieved by the Ethics Committee of the University of Bonn.

All fetuses underwent a detailed scan, using high-resolution ultrasound equipment, before each puncture (each IUT and fetocide), including Doppler sonographic measurements of the arteria umbilicalis, ductus venosus and MCA. To assess fetal anemia the MCA-PSV was measured, with an insonation angle of < 10° and calculated in multiples of the median (MoM) by the formula e^(2.31+0.046/GA)^. Hb values were determined before transfusion or fetocide and were converted to MoM-Hb values. A MoM-Hb value of 0.84–0.65 indicated mild anemia, MoM-Hb values of 0.64–0.55 moderate anemia, and MoM-Hb values of < 0.55 indicated severe anemia. In each case, we sought for reasons of fetal anemia. When Coombs test of maternal blood showed a titer of > 1:32 for Rhesus-antibodies (CcDEe), Rhesus incompatibility was diagnosed. PVB19 infection was confirmed either by PCR of fetal blood/amniotic fluid or by maternal IgM antibodies and sonographic signs for severe anemia. Hydrops fetalis was diagnosed when at least two of the following signs were present, including at least one fetal compartiment: ascites, hydrothorax, pericardial effusion, skin edema, and placentomegaly, cardiomegaly or polyhydramnios. Intrauterine growth restriction (IUGR) was defined as an estimated fetal weight percentile < 10%. The following parameters were analyzed from the fetal blood samples: NT-proBNP (pg/ml) and hemoglobin (g/dl). We assessed the gestational age at the first presentation at our center, at the puncture (each IUT and fetocide) and outcome measures. All fetuses that underwent serial IUTs due to anemia belonged to this group. We excluded anemic fetuses with growth restriction before the first IUT. 10 fetuses in this group have been described in a previous publication [6]. For the puncture, we used a 22G needle, which was guided into the umbilical vein by ultrasound.

### Subjects

Fetal blood sampling was initiated, followed by the IUT. From each blood sample, we primarily measured the fetal hemoglobin to plan the IUT. For some samples, it was not possible to measure the NT-proBNP concentration, if samples clotted or were too small after taking the portion to measure the hemoglobin. To avoid volume overload, we applied no more than 30–50 ml per kilogram estimated fetal weight (without hydrops), using cross-matched, 0 Rhesus-negative and cytomegaly virus-negative, irradiated packed red blood cells. In cases of severe anemia we performed a subsequent IUT 2–5 days later if the hemoglobin concentration was below 10 g/dl at the end of the IUT. Follow-up ultrasound and Doppler scans were achieved on the next day, followed by weekly scans. We collected the number of performed IUTs and the interval between IUTs.

### Controls

Fetocide was performed according to the national legislation, and all fetuses offering conditions that may influence the blood levels of NT-proBNP were excluded, such as cardiovascular dysfunctions (cardiac or thoracic malformations, infections, hydrops), urinary tract malformations, tumors, neuromuscular disorders, growth restriction, pathological Doppler assessment (high resistance in the umbilical artery or ductus venosus) and monochorionic multiple pregnancies. For fetocide, the umbilical vein was punctured by a 22G-needle under ultrasound control and before injection of the potassium chloride, fetal blood was withdrawn for analysis. Fetuses with proven anemia in the blood sample or with suspected anemia, showing increased MCA-PSV were excluded from the control group.

All examined values were tested for normal distribution using the Kolmogorov–Smirnov test. Parameters showing positive skewness, such as NT-proBNP concentration in the subject group, were considered logarithmized. The following influences on blood analysis variables in the control group were examined using multiple linear regressions: Child sex and disease, maternal BMI and age and gestational age. All requirements for a multiple linear regression were met; residuals were tested for normal distribution using the Shapiro–Wilk test. For comparison of means between subgroups, analysis of variance (ANOVA) and the Bonnferoni post-hoc test as well as the Games-Howell post-hoc test were performed. Linear correlations within groups were estimated with the Pearson correlation coefficient. Comparison with previously published values was made by a comparison of mean values and graphically. The course of blood test results of sequential IUTs was explored using a linear mixed model. The significance level was set at *p* < 0.05 for each analysis. We used IBM SPSS Statistics 27.0 for data analyses.

We analyzed the normal blood values of NT-proBNP in fetuses that underwent fetocide and not showing any signs or diseases that could affect the natriuretic system and compared our results to the regression analysis of Merz et al. [15]. Comparing controls and subjects, we analyzed the effect of anemia and the IUT therapy on the NT-proBNP-level. Within the anemic group, we performed two subanalyses. First, we analyzed if there were differences in the circulating NT-proBNP levels, comparing cases with Rhesus incompatibility with those affected by a PVB19 infection and the influence of the diagnosis of fetuses with hydrops. Second, we analyzed the course of the NT-proBNP levels during serial IUT therapy. Reference values for this were calculated by the regression equation of the control group and we added 2 standard deviations as the upper limit.

## Results

**Fig. 1:**
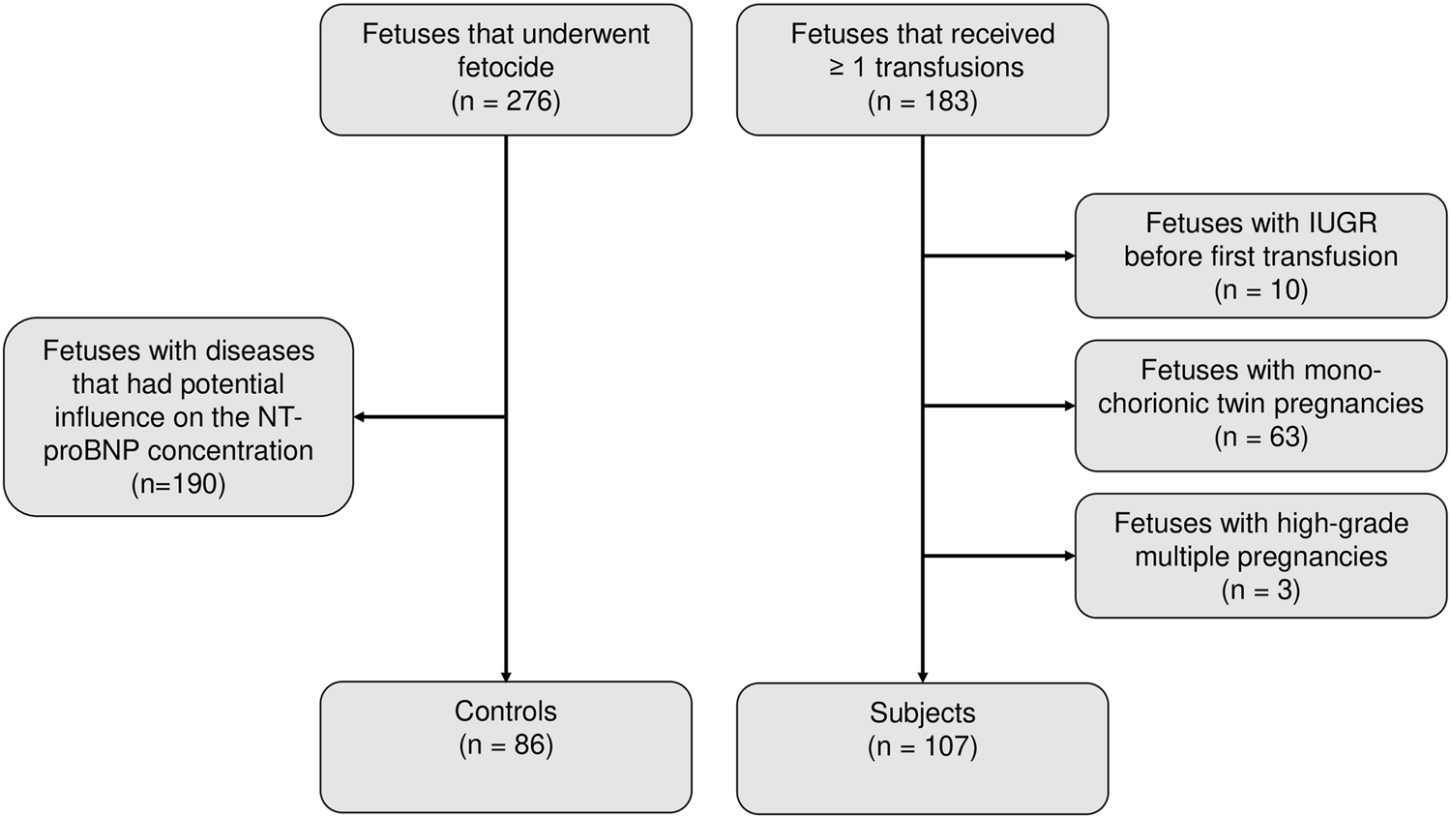
Study flow diagram

86 fetuses, whose blood samples were taken during fetocide, presented to the control group. 190 fetocides had to be excluded, as the fetuses presented with diagnoses that might have had an impact on NT-proBNP levels (see Fig. 1). Reasons for fetocide are listed in Table 1. The subject group consisted of 183 fetuses, which received IUT. We excluded 63 monochorionic twin pregnancies, 3 triplets and 10 IUGRs, leaving *n* = 107 for analysis (see Fig. 1).

**Table 1:** Diagnosed diseases in the group of controls and subjects

Disease				
Controls		Subjects		Number of hydropic fetuses
Skeletal dysplasia	5 (5.8)	Rhesus incompatibility	41 (38.3)	15/41 (36.6)
Neural tube defects	15 (17.4)	Parvovirus B19	29 (27.1)	18/29 (62.1)
Brain Malformations	39 (45.3)	Kell anemia	8 (7.5)	4/8 (50.0)
Trisomy 21	14 (16.3)	Cytomegaly virus	3 (2.8)	3/3 (100.0)
Chromosomal aberrations	2 (2.3)	Chorangioma	3 (2.8)	1/3 (33.3)
Diverse	11 (12.8)	Homozygotic alpha-Thalassemia	1 (0.9)	1 (100.0)
		Congenital dyserythropoetic anemia	1 (0.9)	0
		Trisomy 21	1 (0.9)	1 (100.0)
		Anemia for other or unknown etiology	20 (18.7)	11 (55.0)
**Total**	86 (100)		107 (100)	54/107 (50.0)

Data in number *n* (%)

Reasons for fetal anemia are listed in Table 1. 54 subjects showed hydrops fetalis and were investigated as a subgroup. 41 fetuses had Rhesus incompatibility and 29 had a PVB19 infection.

The median interval between the first and the second IUT was 7 days (*n* = 107, SD = 0.70) and 13 days between the second and the third IUT (*n* = 84, SD = 1.05). The earliest transfusion of the entire sample was at 15 weeks' gestation, and the latest transfusion was at 38 weeks' gestation. The mean value of gestational age at transfusion was 26.8 ± 5.2 weeks. The average number of transfusions was 3.9 transfusions. The median of transfusions per fetus was 3.0 transfusions. There were only two cases that underwent more than 9 transfusions. In both cases, Rhesus incompatibility was present. The first transfusion started at week 17 and 19 and the last transfusion was performed at week 35 and 36 in those cases.

Of the 107 fetuses that underwent serial IUT, 91 were born alive at a median gestational age of 35.1 weeks (SD = 3.7) and a birth weight of 2692 ± 724 on a centile of 49 ± 27. Eleven subjects suffered intrauterine fetal death (IUD). Seven of these fetuses had a PVB19 infection with severe hydrops fetalis. Of those eleven IUDs, five occurred within 24 h after the transfusion. Five children died postnatally. In all of these neonatal deaths (NND), hydrops fetalis was diagnosed during pregnancy. Two of them were delivered at 27 weeks with severe anemia of unknown cause, one at 28 weeks with Cytomegalie virus (CMV) infection, one at 32 weeks with trisomy 21 and one at 21 weeks with multiple chorangiomata. In five cases, no information on the outcome could be collected.

Baseline characteristics, Doppler measurements and blood parameters of subjects and controls, as well as the subgroups Rhesus incompatibility and PVB19 are listed in Table 2. Maternal age and body mass index (BMI) as well as the distribution of the fetal sex were comparable in the groups.

**Table 2:** Basic characteristics and measurements of the patient collective

	Subjects *n* = 107	Subgroup Rhesus Incompatibility *n* = 41	Subgroup PVB19 *n* = 29	Controls *n* = 86
Maternal characteristics				
Age mother [years]^a^	31.4 ± 5.3	32.0 ± 1.5	31.2 ± 5.5	31.4 ± 5.3
BMI mother [kg/m^2^]^a^	26.1 ± 4.5	27.1 ± 4.9	26.2 ± 4.4	27.8 ± 5.7
Gravidity of the mother^a^	3.0 ± 1.6	4.2 ± 1.5	2.7 ± 1.3	2.4 ± 1.3
Mother parity^a^	1.0 ± 1.3	2.0 ± 1.5	1.1 ± 0.9	1.0 ± 1.0
GA at first intervention [week, days]^a^	23.6 ± 6.5	25.3 ± 5.0	19.5 ± 2.6	25.0 ± 3.7
Fetal characteristics				
Male fetuses^b^	51 (47.7)	22 (53.7)	15 (51.7)	38 (44.2)
Female fetuses^b^	53 (49.5)	17 (41.5)	14 (48.3)	48 (55.8)
Dichorionic twins^b^	5 (4.7)	2 (4.9)	1 (3.4)	5 (5.8)
Estimated weight [g]^a^	843 ± 627	1081 ± 664	356 ± 145	704 ± 442
Hydrops fetalis^b^	54 (50.5)	15 (36.6)	18 (62.1)	0
Outcome				
Live births^b^	91 (80.4)	37 (90.2)	22 (75.9)	0
Birth weight (BW) [g]^a^	2692 ± 724	2658 ± 584	2950 ± 1078	
Percentile of BW^b^	49 ± 27	53 ± 27	36 ± 25	
Intrauterine death (IUD)^a^	11(10.3)	0	7 (24.1)	
No information^b^	5 (4.7)	4 (9.7)	0	

^a^Data in mean (M) ± standard deviation (SD)

^b^Data in number *n* (%)

**Fig. 2:**
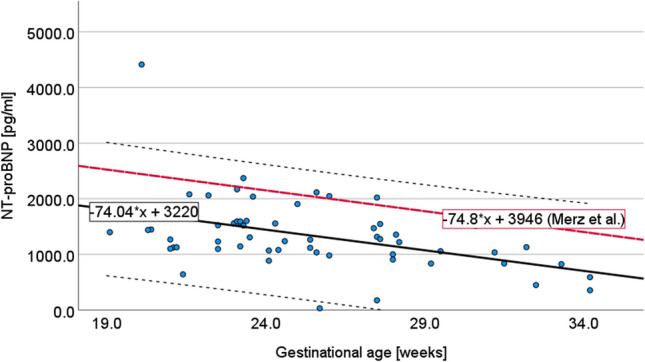
Correlation of NT-proBNP concentration [pg/ml] in fetal blood of controls with gestational age [weeks] of mothers at the time of collection, *R* = − 74.04, *T* = − 3.65, *p* = 0.001. The regression analysis of Merz et al. is depicted for comparison

The NT-proBNP concentration in the control group showed a mean of 1339 ± 639 pg/ml. There was a significant decline in NT-proBNP concentration with advancing gestational age (*p* = 0.001), showing an average decrease of 74 pg/ml per week of gestation. Overall, the mean dropped from 1813 pg/ml at 19 weeks to 702 pg/ml at week 34 (see Fig. 2). There was one outlier in the group with an NT-proBNP value of 4413 pg/ml. This fetus had a normal Hb and was not anemic. Fetocide was performed for open spina bifida. The different fetal diagnoses, maternal age and BMI showed no influence on NT-proBNP concentration in the multiple linear regression (*p* > 0.05). Only the male sex showed a slightly increased NT-proBNP concentration compared to the female sex (*p* = 0.006, *β* = 0,34, *R* = 431). However, the strength of this influence was lower, measured by a standardized coefficient ((*β*) = 0.3), than the influence of gestational age (*β* = 0.5).

**Fig. 3:**
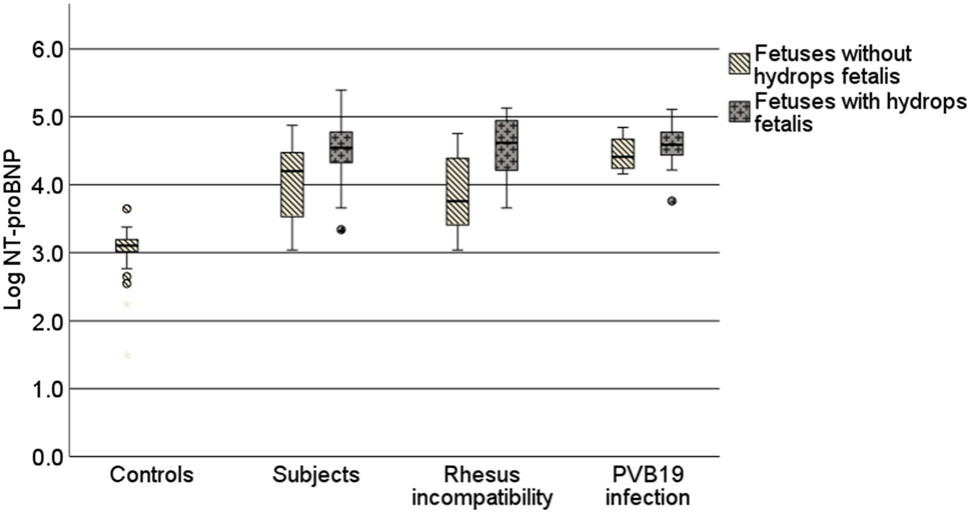
Median (*Q*1–*Q*3) log NT-proBNP concentration [pg/ml] in fetal blood of controls, subjects and subgroups. Fetuses with and without hydrops fetalis are presented separately in each group

In the group of subjects, there was a strong correlation between the severity of fetal anemia (MoM-Hb) and both, NT-proBNP values (*R* = − 0.64, *p* < 0.001) as well as MoM-MCA-PSV values (*R* = − 0.50, *p* < 0.001). ANOVA showed significant differences in the NT-proBNP levels (*F* = 48.5, *p* < 0.001), MoM-Hb values (*F* = 81.62, *p* < 0.001) and MoM-MCA-PSV values (*F* = 68.81, *p* < 0.001), comparing controls and subjects, subgroups of fetuses with PVB19 infection and fetuses with Rhesus incompatibility as well as fetuses with and without hydrops fetalis in the fetal blood before the start of therapy, compared to the controls at the time of fetocide (see Fig. 3). The subgroup of anemic fetuses with PVB19 infection showed the highest blood levels of NT-proBNP concentration, compared to the control group (*p* < 0.001) and compared to the subgroup of Rhesus incompatibility (*p* = 0.026) (see Fig. 3). But the mean value of fetuses with Rhesus incompatibility was also significantly higher compared to the control group (*p* < 0.001). MoM-MCA (*p* = 0.330) and MoM-Hb levels (*p* = 0.892) did not show a significant difference between fetuses with Rhesus incompatibility and fetuses with PVB19 infection. Moreover, fetuses with hydrops showed significantly higher NT-proBNP levels compared to anemic fetuses without hydrops (*p* = 0.006) and also compared to controls (*p* < 0.001). Before the start of therapy, subjects with hydrops fetalis showed a mean NT-proBNP concentration of 52,946 ± 54,777 pg/ml, whereas fetuses without hydrops showed a mean of 21,959 ± 22,004 pg/ml. Those observations could also be made for MoM-Hb (*p* < 0.001) and MoM-MCA (*p* = 0.049) when comparing the hydropic fetuses to non-hydropic fetuses.

**Fig. 4:**
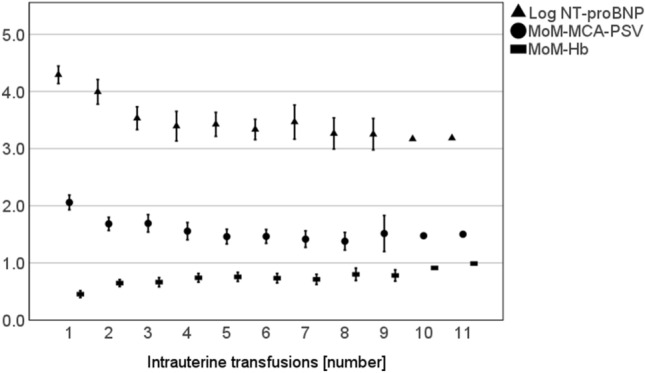
Longitudinal presentation of measurements before each intrauterine transfusion [number] showing the mean ± 95%CI of logarithmized NT-proBNP concentration

During therapy, NT-proBNP (*β* = − 0.14, *p* < 0.001, 95%CI = − 0.17 to − 0.11) and MoM-MCA-PSV (*β* = − 0.09, *p* < 0.001, 95%CI = − 1.1 to − 0.07)) values significantly decreased, while the MoM-Hb (*β* = 0.05, *p* < 0.001, 95%CI = 0.04 to 0.06) values significantly increased (see Fig. 4 and Table 3). NT-proBNP measurement was not possible in all samples before each IUT. Table 3 displays the number of measured values. The greatest change in all parameters occurred between the first and second transfusion. The median of NT-proBNP decreased 47% between the first and second IUT (95%CI = − 63% to − 31%) and 36% between the second and third IUT (95%CI = − 62% to − 10%). Based on the GA-dependent values obtained, only 5% of the measured NT-proBNP concentrations were within the physiological range before the first IUT. After the third IUT, it was 63% of the measured values (*n* = 14). Thus, more fetuses had physiological NT-proBNP concentrations than pathological NT-proBNP concentrations. MoM-Hb values increased from a median of 0.42 (*Q*1–*Q*3 = 0.24–0.58) to a median of 0.78 (*Q*1–*Q*3 = 0.55–0.84) from the first to second IUT. MoM-MCA-PSV values decreased from a median of 2.12 (*Q*1–*Q*3 = 1.78–2.36) to 1.59 (*Q*1–*Q*3 = 1.33–1.95) from the first to second IUT. Overall, levels of MoM-MCA-PSV and MoM-Hb remained pathological before each transfusion. See Table 3 for the measured values before each IUT.

**Table 3:** Measurements of different parameters in controls subjects and subgroups before feticide (FC) (controls) or each intrauterine transfusion (IUT) (subjects)

	NT-proBNP [pg/ml]	MoM-MCA-PSV	MoM-Hb
FC			
Controls *n* = 86	1399 ± 639 [58]	1.00 ± 0.21 [63]	1.04 ± 0.10 [54]
IUT 1			
Subjects *n* = 107	37,452 ± 44,205 [54]	2.12 ± 0.42 [107]	0.43 ± 0.21 [100]
Rhesus Incompatibility *n* = 41	27,937 ± 37,680 [23]	2.05 ± 0.42 [41]	0.44 ± 0.21 [40]
PVB19 *n* = 29	46,420 ± 36,982 [14]	2.22 ± 0.39 [29]	0.40 ± 0.20 [24]
IUT 2			
Subjects *n* = 80	28,490 ± 57721 [39]	1.65 ± 0.41 [80]	0.66 ± 0.18 [79]
Rhesus Incompatibility *n* = 36	9366 ± 14,261 [16]	1.59 ± 0.36 [36]	0.68 ± 0.21 [37]
PVB19 *n* = 15	33,677 ± 20,504 [8]	1.71 ± 0.39 [15]	0.63 ± 0.18 [14]
IUT 3			
Subjects *n* = 56	6453 ± 7793 [28]	1.60 ± 0.35 [56]	0.66 ± 0.20 [52]
Rhesus Incompatibility *n* = 35	5078 ± 6336 [16]	1.59 ± 0.40 [35]	0.67 ± 0.20 [32]
PVB19 *n* = 4	22,603 ± 16,249 [2]	1.69 ± 0.36 [4]	0.71 ± 0.12 [4]
IUT 4			
Subjects *n* = 44	6710 ± 11,933 [22]	1.59 ± 0.44 [44]	0.70 ± 0.19 [43]
Rhesus Incompatibility *n* = 28	7882 ± 14,152 [13]	1.60 ± 0.50 [28]	0.69 ± 0.21 [28]

Values in mean ± SD [measured values]

**Fig. 5:**
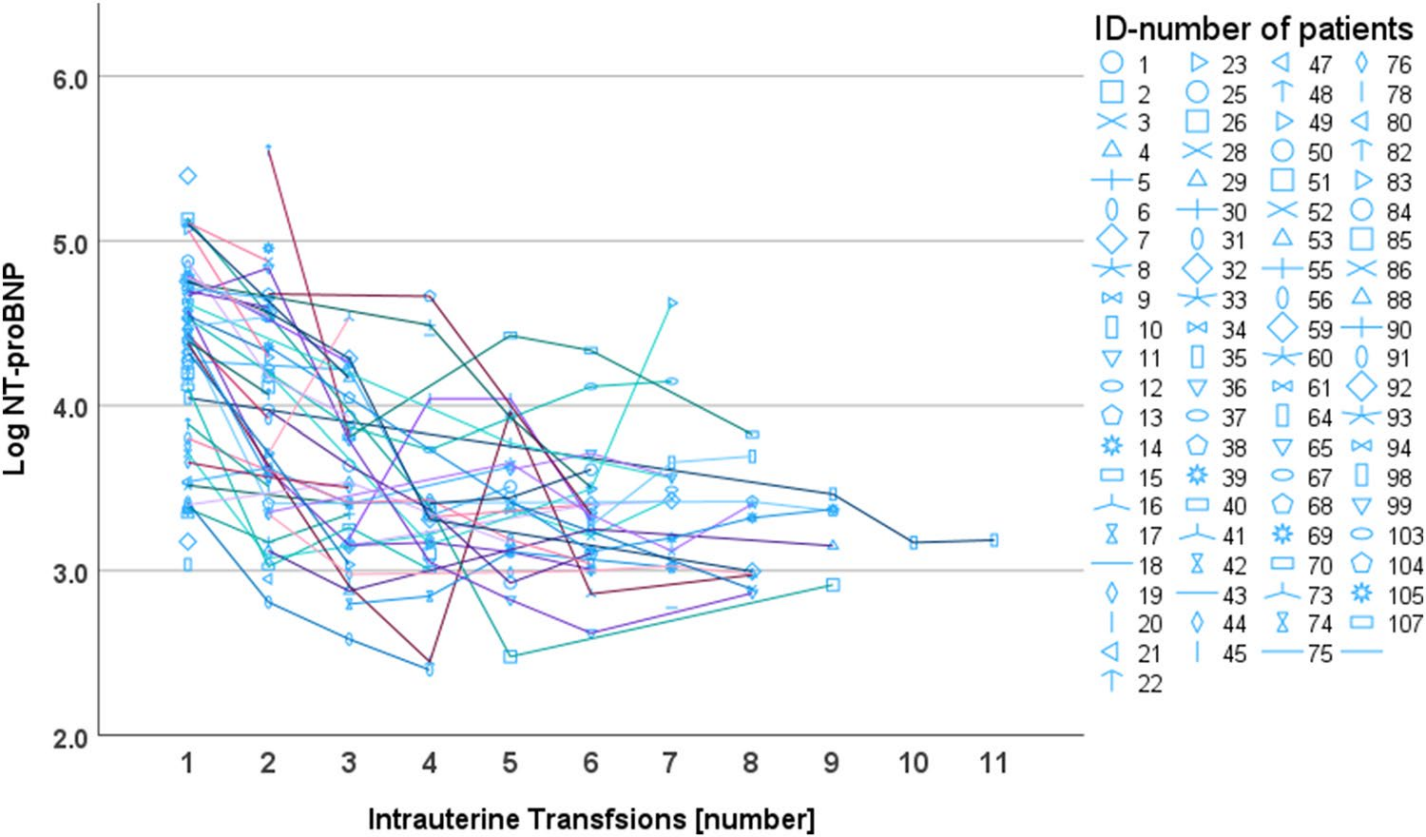
Longitudinal plot of logarithmized NT-proBNP concentration in a spaghetti plot. Each color represents one patient (ID-number of patients) over the therapy period (number of intrauterine transfusions). If NT-proBNP measurement was missing for an IUT the line connects to the priorly measured value

There were seven cases in which NT-proBNP concentration did not decrease after each transfusion. All fetuses were delivered alive. In 6 of these cases, the fetuses developed hydrops fetalis during the course of therapy (see Fig. 5).

Throughout the course of IUT therapy, the differences in parameters described at the beginning remained (see Fig. 4). Hydropic fetuses showed higher levels of NT-proBNP in the cord blood than non-hydropic fetuses (*β* = 0.34, *p* = 0.001, 95%CI = 0.54 to 0.15). MoM-Hb-values were significantly lower during IUT therapy in hydropic fetuses (*β* = − 0.11, *p* < 0.001, 95%CI = 0.06 to 0.17). Only MoM-MCA-PSV did not show significant differences during therapy (*p* = 0.279). Fetuses with PVB19 infection showed significantly higher NT-proBNP levels compared to fetuses with Rhesus incompatibility during the first three IUTs (*β* = 0.58, *p* < 0.001, 95%CI = 0.88 to 0.27).

## Discussion

Because of its long half-life and high thermostability, NT-proBNP is an established marker for cardiac function in adults. In contrast to image-based diagnostics, there is no inter- or intraobserver variability. The correlation between cardiac stress and NT-proBNP concentration also appears to be strong in the fetal organism [7, 11, 16, 17]. Although MCA-PSV measurement shows high reliability for anemia diagnosis, it cannot reliably predict the occurrence of hydrops fetalis [18] and the severity of cardiac compromise and is affected during IUT therapy by transfused adult erythrocytes [19]. However, fetal vasoconstriction could be verified in MCA Doppler measurements after intrauterine transfusion, shown as a biphasic contour of systolic blood flow, indicating a pulse wave reflection due to vasoconstriction [20].

Physiological reference values of NT-proBNP concentration in fetal blood are difficult to define due to ethical considerations of the invasive procedure and different analytical methods [21]. So far, there are only very few studies that deal with this topic. Fortunato et al. [22] performed blood sampling in a cohort of 22 fetuses in the second trimester. The mean NT-proBNP concentration was 2308 pg/ml. Exclusion criteria were multiple pregnancies, severe fetal anomalies, and abnormal karyotypes. Walther et al. [23] calculated an average NT-proBNP concentration of 1052 ± 182 pg/ml in the fetal blood of 9 fetuses. Blood samples were taken on the suspicion of fetal infection in the second trimester, which was not confirmed in the blood analysis. Both studies had a small number of cases and were limited to values before 25 weeks of gestation. Merz et al. [10] determined a mean NT-proBNP concentration in 59 fetuses of 1998 pg/ml (± 2SD = 242–3754) between 20 and 34 weeks of gestation. Exclusion criteria included structural malformations of the cardiovascular and urogenital systems and conditions with potential influence on NT-proBNP concentration. In our study, the average measured NT-proBNP concentration was 1339 ± 639 pg/ml between 20 and 35 weeks of gestation (*n* = 58), so it correlates with the previous data, using the same analytical procedure. Besides Merz et al. our results represent the largest sample size for normal NT-proBNP values in accordance with a gestational age up to 35 weeks of gestation. Our regression analysis showed a significant decrease in NT-proBNP concentration with increasing gestational age (− 74.04*GA + 3220 pg/ml, *p* = 0.001), which correlates well to the regression calculated by Merz et al. (− 74.8*GA + 3946 pg/ml, *p* = 0.012) (see Fig. 2). NT-proBNP concentrations in umbilical cord samples in term pregnancies are significantly lower than values measured in the second trimester [22, 24].

Compared to adult reference values (0–10 pg/ml) and NT-proBNP blood concentration of pregnant women, fetal blood levels are significantly higher. Placental exchange during pregnancy and lack of renal elimination were excluded as causes [8, 22, 25]. The higher NT-proBNP concentration appears to result from intrinsic fetal production of this hormone [15]. Natriuretic peptides are involved in the development of various organ systems during the fetal period and are mainly responsible for the growth of cardiovascular tissue. Animal experiments have shown that both, ANP and BNP, can suppress the growth of cardiac fibroblasts and regulate the growth of cardiac tissue [8, 26]. From mid-pregnancy onwards, the natriuretic peptide system appears to start its postnatal function and to be involved in the control of blood pressure and salt concentration via myocardial stress [8]. Thus, the prenatally increased NT-proBNP concentration may be explained as an expression of both, fetal cardiovascular maturation and high cardiac volume load in the fetal circulation. Thus, the decrease during the course of pregnancy could be explained by cardiac maturation and the decrease in left ventricular afterload [10]. The correlation of left ventricular volume load and NT-proBNP concentration is also observed in the cord blood of neonates in the first days of life [27]. Pathological elevation of NT-proBNP concentrations in fetal blood is associated with cardiovascular dysfunction of different causes [28]. The presence of anemia increases cardiac output due to decreased blood oxygen and leads to myocardial stress with activation of the natriuretic peptide system [29]. BNP in particular is thought to play a critical role in cardiac remodeling under hypoxic myocardial injury [25]. The present study considered the NT-proBNP concentration in anemic fetuses, focusing on those with Rhesus incompatibility and PVB19 infection, as well as on the status of cardiac decompensation in hydrops fetalis. Our results confirmed that NT-proBNP concentration is pathologically elevated in fetal anemia and increases with anemia severity (*R* = − 0.64, *p* < 0.001), regardless of the cause of anemia. Fetuses with Rhesus incompatibility are known to have increased NT-proBNP concentrations in fetal blood [14, 23, 29].

In our study, fetuses with a PVB19 infection showed the highest NT-proBNP levels (see Fig. 3), whereas their MoM-Hb values and MoM-MCA-PSV values showed no significant difference compared to fetuses with Rhesus incompatibility (see Table 3). It has to be noticed, that the median of the first IUT was 6 weeks earlier in fetuses with PVB19 infection than in those with Rhesus incompatibility and we know that NT-proBNP levels decrease with progressing pregnancy (see Fig. 2). It could also be stated that fetuses with PVB19 infection suffer a more severe cardiac compromise than fetuses with Rhesus incompatibility at the same anemia severity. These results are in contrast to the work of Merz et al. [6], who found no effect of PVB19 infection on NT-proBNP concentration (*n* = 8). The different results could be due to their smaller sample size. Further studies are needed, analyzing NT-proBNP concentration in the blood of fetuses with PVB19 infection, as no further studies could be found in our literature search. An explanation of the high NT-proBNP levels of PVB19-infected fetuses could be the associated PVB19 myocarditis, which results from a viral infection of myocardial cells [30–32].

It has to be mentioned that in our cohort, there was a high prevalence of fetuses with hydrops fetalis in the PVB19 subgroup (62%), which is higher than in other reported studies with a prevalence of up to 24% [33] and 43% in a recent study of our group [34]. This may have resulted from the selection of fetuses, as only fetuses requiring therapy were included. In addition, the average gestational age in the PVB19 subgroup was less than 20 weeks’ gestation, thus increasing the risk of hydrops fetalis [35]. In fetuses with PVB19 infection, the prevalence of hydrops fetalis is known to be high, even in less severe anemia, as PVB19 infection prevalence is high at earlier weeks of gestation when diastolic and systolic cardiac function is still limited, and depending on the venous pressure, lead to hydrops more frequently [30, 36–38]. Studies on NT-proBNP concentration in hydropic fetuses with PVB19 infection are not available.

Fetal hydrops develops in fetuses with severe anemia (Hb < 4–7 g/dl) when compensatory mechanisms are exhausted [39, 40]. An increased NT-proBNP concentration has already been demonstrated in fetuses with anemia when hydrops was present [6], which was also significantly shown in our results (see Fig. 3). Yarav et al. [29] presented high NT-proBNP concentrations in 10 hydropic fetuses with Rhesus incompatibility, showing an increasing concentration with increasing severity of hydrops fetalis.

IUT therapy improves survival rates in fetuses with anemia, even though the cardiac load is temporarily increased by the transfused blood [41]. Walther et al. [23] showed a 13% short-term increase in NT-proBNP concentration during IUT and explained this by the transfusion-related volume load. Other studies showed a decrease in NT-proBNP concentration before subsequent IUTs in fetuses with Rhesus incompatibility [14, 29]. Merz et al. [6] further described a normalization of NT-proBNP concentration after three IUTs, while increased MCA-PSV and low Hb remained as signs of anemia (*n* = 27) Our results share this observation (see Fig. 4). Our use of reference values, which were calculated and adjusted for gestational age, increases the reliability of detecting pathological concentrations. Before the first IUT, only 5% of the measured NT-proBNP concentrations were within the reference range, after the third IUT, 64% of these values were within the reference range, while the measured MoM-MCA-PSV values and the MoM-Hb values remained pathological before each IUT (see Table 3). It appears that myocardial distress decreased, despite the transfused volume and the presence of anemia. A major factor was certainly the increase and transient normalization for a longer period in hemoglobin after transfusion. Although adult erythrocytes have a poorer oxygen-binding curve than fetal erythrocytes, the latter have a less rigid cell membrane and smaller volumes. In addition, cardiac remodeling as an adaptation of fetal cardiomyocytes to the hypoxic state may have occurred [6]. In fetal sheep, an increase in myocardial mass and myocardial vascularization were observed during the course of anemia, reflecting the high adaptability of cardiomyocytes [42]. In accordance with the results of Merz et al. [6] and Luterek et al. [14] by using the specific GA-dependent reference values, our results show a decrease in NT-proBNP concentration and cardiac unloading during serial IUTs.

Before the first transfusion, the NT-proBNP concentration reliably reflects the extent of cardiac stress or overload, as shown in the present study. NT-proBNP concentration also appears to reliably describe therapeutic success in terms of cardiac unloading. Furthermore, the correlation between the presence of hydrops fetalis and a high NT-proBNP concentration is high. Since the presence of hydrops is the most important prognostic factor influencing survival after IUT therapy, NT-proBNP concentration can indirectly be used to predict outcome. Merz et al. [6] showed that NT-proBNP < 10,000 ng/l has a negative predictive value of 100% for the identification of fetal hydrops, with a positive predictive value of 44%.

There were seven cases in our cohort in which NT-proBNP concentration did not decrease after each transfusion. All fetuses were delivered alive. However, in 6 of these cases, hydrops persisted with distinct anemia-induced cardiomyopathy for a long period during the course of IUT treatment. This strengthens the hypothesis that NT-proBNP increases under the development of hydrops fetalis.

In an invasive therapy such as sequential IUT therapy, the implemented determination of NT-proBNP concentration may allow an assessment of the cardiac status and may help to detect the risk of developing hydrops fetalis due to cardiac decompensation in the future [15].

## Conclusion

NT-proBNP is an established biomarker for cardiac dysfunction used in adult and pediatric medicine and has been extended to fetal life. NT-proBNP concentrations are described to be distinctly higher in the prenatal than in the postnatal period. We could show, that the physiological concentration of this marker decreases with ongoing pregnancy and established GA-dependent normal values in cord blood. Anemia increases the workload of a fetal heart, so we found elevated concentrations in fetal blood, which correlated well with the degree of anemia. Highest levels of NT-proBNP values were found in cases with heart failure, shown by hydrops fetalis. For the same extent of anemia, PVB19-induced anemia showed higher NT-proBNP concentrations than Rhesus incompatibility, maybe due to a virus-associated myocarditis, but also because PVB19-associated anemia was diagnosed at an earlier gestational age with higher levels per se. In both groups, IUT treatment resulted in a significant decrease of the NT-proBNP concentration, whereas Hb and MCA-PSV measurements remained abnormal. By indicating the extent of cardiac stress NT-proBNP may be a useful marker in the management of fetal anemia and IUT monitoring, especially in cases at risk for hydrops fetalis.

## Data Availability

Data were collected from patient files, Viewpoint and ORBIS and are not publicly available to preserve individuals' privacy under the European General Data Protection Regulation.
